# Role of the lesion scar in the response to damage and repair of the central nervous system

**DOI:** 10.1007/s00441-012-1336-5

**Published:** 2012-02-25

**Authors:** Hitoshi Kawano, Junko Kimura-Kuroda, Yukari Komuta, Nozomu Yoshioka, Hong Peng Li, Koki Kawamura, Ying Li, Geoffrey Raisman

**Affiliations:** 1 Laboratory of Neural Regeneration, Tokyo Metropolitan Institute of Medical Science, Setagaya City, Tokyo 156-8506 Japan; 2 Department of Human Anatomy, College of Basic Medical Sciences, China Medical University, Shenyang, 110001 China; 3 Spinal Repair Unit, Institute of Neurology, University College London, Queen Square, London, WC1N 3BG UK

**Keywords:** Traumatic injury, Central nervous system, Glial scar, Fibrotic scar, Blood–brain barrier, Axonal regeneration

## Abstract

Traumatic damage to the central nervous system (CNS) destroys the blood–brain barrier (BBB) and provokes the invasion of hematogenous cells into the neural tissue. Invading leukocytes, macrophages and lymphocytes secrete various cytokines that induce an inflammatory reaction in the injured CNS and result in local neural degeneration, formation of a cystic cavity and activation of glial cells around the lesion site. As a consequence of these processes, two types of scarring tissue are formed in the lesion site. One is a glial scar that consists in reactive astrocytes, reactive microglia and glial precursor cells. The other is a fibrotic scar formed by fibroblasts, which have invaded the lesion site from adjacent meningeal and perivascular cells. At the interface, the reactive astrocytes and the fibroblasts interact to form an organized tissue, the glia limitans. The astrocytic reaction has a protective role by reconstituting the BBB, preventing neuronal degeneration and limiting the spread of damage. While much attention has been paid to the inhibitory effects of the astrocytic component of the scars on axon regeneration, this review will cover a number of recent studies in which manipulations of the fibroblastic component of the scar by reagents, such as blockers of collagen synthesis have been found to be beneficial for axon regeneration. To what extent these changes in the fibroblasts act via subsequent downstream actions on the astrocytes remains for future investigation.

## Introduction

After damage to the central nervous system (CNS) of adult mammals, regeneration of transected axons barely occurs. There is a growing view that severed central axons are capable of regeneration and that the failure to regenerate is due to the blocking effect of the scar formed at the lesion site. This scar consists in both glial (mainly astrocytic) and fibrotic components (Fitch and Silver 2008) and may have its inhibitory effects both by the production of inhibitory molecules and by the physical abrogation of aligned pathways for regenerating axons to cross the lesion. At the same time, the scarring process fulfils the vital functions of restoring the blood–brain barrier (BBB) and limiting the damage to the site of injury. This review will cover a number of recent studies in which manipulations of the fibroblastic component of the scar by reagents, such as blockers of collagen synthesis, have been found to be beneficial for axon regeneration.

Various kinds of inhibiting factors that are upregulated around the lesion site have been postulated to prevent the regrowth of severed axons beyond the lesion site. These include molecules of the chondroitin sulfate proteoglycan (CSPG) family (for review, see Asher et al. 2001; Morgenstern et al. 2002; Tan et al. 2005), tenascin (McKeon et al. 1991), semaphorin 3A (Pasterkamp et al. 1999), myelin-associated molecules (reviewed by Zörner and Schwab 2010) and subtypes of the Eph receptors and their ligands ephrins (reviewed by Goldshmit et al. 2006). These molecules have axonal growth-repelling activities in vitro and play important roles in axon guidance in the developing CNS. Attempts to eliminate the molecules or neutralize the inhibitory effect have been reported to enhance axonal regeneration in the damaged brain and spinal cord (Bradbury et al. 2002; Goldshmit et al. 2006; Kaneko et al. 2006; Moon et al. 2001).

**Fig. 1:**
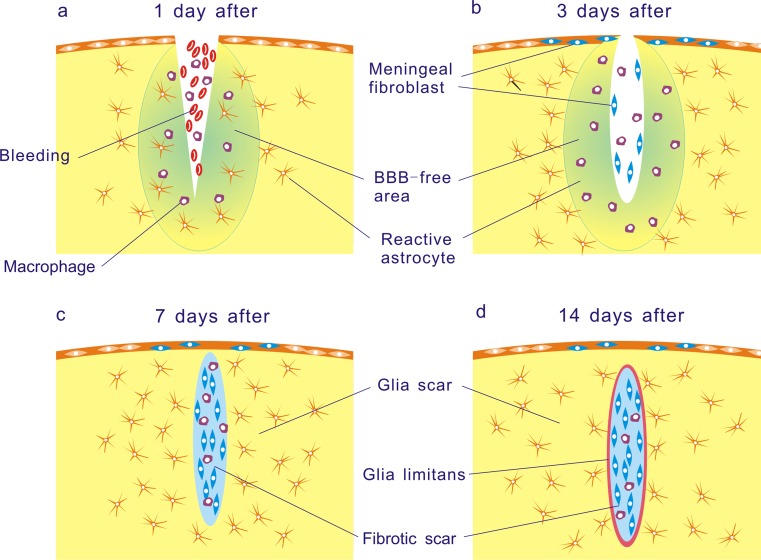
Schematic drawings represent the process of the lesion scar formation in the mouse brain. One day after traumatic CNS injury, the BBB is disrupted and macrophages infiltrated the BBB-free area. **a** Upregulation of GFAP immunoreactivity in reactive astrocytes is already observed. **b** Three days after the injury, reactive astrocytes significantly increase around the lesion site, but they are absent from the lesion center where the BBB is destroyed. Fibroblasts intrude from the damaged meninges to the lesion site. **c** By 1 week after injury, fibroblasts actively proliferate and secrete ECMs to form the fibrotic scar. Reactive astrocytes re-occupy the surrounding area of the lesion site and BBB-free area around the lesion site is eliminated. **d** At 2 weeks after, processes of reactive astrocytes seal the lesion site to form a glia limitans

The molecular changes in the glial and fibrotic scar are closely related with the tissue repair process of the CNS lesion site. Following CNS injury, bleeding occurs and the BBB is broken down. The infiltration of blood proteins such as thrombin (Nishino et al. 1993) and fibrinogen (Ryu et al. 2009) triggers the inflammatory reaction. At the same time, hematogenous cells including leukocytes, macrophages and lymphocytes also invade from the lesion site to the surrounding neural tissue and secrete various cytokines and chemokines (Donnelly and Popovich 2008; Merrill and Benveniste 1996). Under the influence of these factors, astrocytes, microglia and oligodendrocyte progenitor cells are activated and constitute the glial scar around the lesion site. On the other hand, from several days after injury, fibroblasts intrude from the damaged meninges to the lesion site, proliferate and secrete extracellular matrix molecules (ECMs) including type IV collagen (Type IV collagen), fibronectin and laminin to form the fibrotic scar (Fig. 1). The composition and arrangements of cells in these lesion scars are postulated to play important roles for the protection of damaged tissue, re-establishment of the BBB and isolation of the lesion site from the surrounding neural tissue (Berry et al. 1983; Mathewson and Berry 1985; Maxwell et al. 1990a; for review, see Shearer and Fawcett 2001). Simultaneously, the cells of these scars express the above-mentioned axonal growth-inhibiting molecules, which are believed to prevent the axonal regeneration and functional recovery in the injured CNS.

## Glial scar and tissue repair

The formation of a glial scar is generally referred to reactive gliosis. After CNS injury, astrocytes, microglia and glial progenitor cells around the lesion site are activated and increase in number. They express and release various bioactive substances, which play important roles in tissue repair processes including inflammation, BBB repair and neural protection (Rolls et al. 2009). Above all, there is a major rearrangement of the anatomical structure. Immediately after CNS injury, resident astrocytes become hypertrophic and extend thick processes together with increased glial fibrillary acidic protein (GFAP) immunoreactivity. Upregulation of GFAP immunoreactivity in astrocytes is observed as early as 1 day after injury (Fig. 1a). Recent imaging shows that astrocytic processes react within hours of the injury (Sibson et al. 2008). A recent study suggests that glial progenitors around the lesion site also generate reactive astrocytes (Yoshioka et al. 2012). At 3–5 days after the injury, there is a significant increase in the numbers of reactive astrocytes around the lesion site but they are absent from the lesion center where the BBB is broken down (Fig. 1b). Concomitant with the accumulation of reactive astrocytes surrounding the lesion site by 1 week after injury (Fig. 1c), the BBB-disrupted area becomes confined as a result of reactive astrocytes enclosing the lesion site to form a glia limitans (Fig. 1d; Mathewson and Berry 1985; Yoshioka et al. 2010).

The astrocytic sealing of the lesion site contributes to homeostatic functions including maintenance of extracellular ion and fluid balance, clearance of extracellular glutamate, water transport, production of pro- or anti-inflammatory cytokines and chemokines, production of growth factors and free radical scavenging (Rolls et al. 2009; Sofroniew 2009). Recent studies using gene manipulation to suppress reactive gliosis showed that in the traumatic CNS injury reactive astrocytes played roles essential in prevention of neuronal death, repair of the destructed BBB and restriction of post-injury inflammation (Bush et al. 1999; Faulkner et al. 2004; Herrmann et al. 2008; Okada et al. 2006; Pekny et al. 1999). The vasculature of the CNS constructs a highly specialized biological interface: the BBB, which helps to maintain homeostasis within the CNS. To accomplish this, brain capillaries possess extensive tight junctions between endothelial cells and the astrocytic processes directly invest the endothelia. The ability of endothelial cells to form a BBB is not intrinsic to these cells but instead is induced by astrocytes. Grafts of astrocytes induce BBB-like properties in peripheral endothelia in vivo (Janzer and Raff 1987) and fetal astrocytes induce various properties of the BBB in cultured endothelial cells (Hayashi et al. 1997). In mice deficient for both GFAP and vimentin intermediate filaments in astrocytes, glial formation was impaired and bleeding occurred frequently after brain and spinal cord injury (Pekny et al. 1999). A lack of these intermediate filaments in perivascular astrocytes decreases the mechanical strength of blood vessels, suggesting that astrocytes normally support the structure of the blood vessels in the CNS. The genetic ablation of proliferating reactive astrocytes from the injured CNS also causes the failure of BBB repair (Bush et al. 1999; Faulkner et al. 2004).

These studies also demonstrate that the glial scar plays a crucial beneficial role in the restriction of leukocyte spreading after CNS trauma. Increased invasion of leukocytes was observed in mice with the genetic ablation of dividing reactive astrocytes from the injured CNS (Bush et al. 1999; Faulkner et al. 2004). In mice with the genetic depletion of Stat3, a mediator of cytokine action, astrocyte migration toward the lesion site is disrupted and leukocytes abnormally spread (Herrmann et al. 2008; Okada et al. 2006). Limiting the infiltration of inflammatory leukocytes and restoring the BBB are considered to reduce the post-traumatic secondary injury after spinal cord injury (Donnelly and Popovich 2008).

The earlier concept (e.g., Banker 1980) that astrocytes provide nutritive, neurotrophic and other supportive functions for neurons has been greatly strengthened by the recent demonstration that the energy metabolism of neuronal mitochondria is dependent on lactate energy supplied by the adjoining astrocytes (Herrero-Mendez et al. 2009; Tsacopoulos and Magistretti 1996).

## Glial scar as an impediment for axonal regeneration

Closing off the lesion site and re-establishment of a glia-pial barrier produce a re-duplicated tangle of astrocytic processes, that completely abrogate any pathways which regenerating axons might have used to cross the lesion (Raisman and Li 2007). The scar further contributes to the failure of axonal regeneration through the axonal growth-inhibiting property of ECMs produced by reactive astrocytes (reviewed by Asher et al. 2001; Höke and Silver 1996). Among them, CSPGs produced by glial scar have been considered as major impediments for axonal regeneration (for review, see Carulli et al. 2005 ; Morgenstern et al*.* 2002; Silver and Miller 2004; Yiu and He 2006). Phosphacan, neurocan, brevican and NG2 have been reported to have an axonal growth-inhibiting property (Dou and Levine. 1994; Friedlander et al. 1994; Milev et al. 1994; Yamada et al. 1997) and increase markedly after CNS injury (Tang et al. 2003). The inhibitory property of CSPGs may reside in the chondroitin sulfate (CS) side chains, since the administration of chondroitinase ABC (ChABC), a CS-degrading enzyme, into the lesion site, effectively promotes regeneration of severed axons in the nigrostriatal ascending (Moon et al. 2001) and spinal descending (Bradbury et al. 2002) pathways. In addition, administration of a DNA enzyme whose target is the mRNA of a critical enzyme, xylotransferase-1, which initiates glycosylation of the protein backbone of CSPGs, also promotes axonal regeneration in the injured rat spinal cord (Grimpe and Silver 2004). Recently, a transmembrane tyrosine phosphatase, PTPσ, was reported to act as a receptor for CS and mediate the axonal growth-inhibiting signal of CSPGs (Shen et al. 2009).

Others have questioned to what extent the glial scar and CSPGs are inhibitory to axon regeneration. NG2 proteoglycan is a major CSPG upregulated after CNS injury and considered as a potent inhibitor of axonal growth in the glial scar (reviewed in Tan et al. 2005). Regenerating axons have been reported to pass through the glial scar (Camand et al. 2004), an area abundant in NG2 proteoglycan (Jones et al. 2003). More recently, after the spinal cord lesion, growth of serotonergic axons was shown to be suppressed in the scar tissue in mice lacking NG2 (de Castro et al. 2005). Finally, NG2 cells, generally referred as oligodendrocyte precursor cells, which are abundant in the glial scar, do not inhibit but promote axonal growth even in the presence of elevated level of NG2 (Yang et al. 2006).

Experiments aimed at genetic suppression of the glial scar have been introduced to evaluate axonal regeneration after CNS injury. The double genetic deletion of GFAP and vimentin, cytoskeletal proteins of astrocytes, has been reported to promote axonal sprouting and functional recovery after spinal cord injury (Menet et al. 2003). In contrast, a line of evidence indicates that genetic ablation of reactive astrocytes prevents the glial scar formation in damaged CNS but fails to promote axonal regeneration (Herrmann et al. 2008; Okada et al. 2006). Although randomly oriented nerve fibers were increased along the wound margin in mice deleted with astrocytes, they did not extend for long distances (Bush et al. 1999). Failure of the axonal regeneration in mice with glial scar deletion may be attributed to the enlarged inflammation in these animals as mentioned in the previous chapter. Although glial scar may be an obstacle to axonal regeneration in damaged CNS, suppression of the glial scar formation cannot be useful for the treatment of traumatic injury in the CNS.

## Fibrotic scar and tissue repair

After CNS trauma, fibroblasts invade the lesion site, proliferate and secrete ECMs, such as Type IV collagen, fibronectin and laminin. The invading fibroblasts cooperate with the astrocytes to lay down a continuous basal lamina on the outer-facing astrocytic surface, thus re-establishing the glia-pial barrier referred to as the glia limitans (Mathewson and Berry 1985; Maxwell et al. 1990a; Shearer and Fawcett 2001). Morphological evidence suggests that the fibrotic scar appears to seal off the lesion site and encloses leukocytes infiltrated after brain injury (Berry et al. 1983; Maxwell et al. 1990a). However, suppression of fibrotic scar formation with an administration of iron chelator 2,2^′^-dipyridyl (DPY), an inhibitor of Type IV collagen synthesis (Ikeda et al. 1992), or with suppression of the function of transforming growth factor-β (TGF-β), significantly reduces the recruitment of inflammatory leukocytes (Logan et al. 1999b; Yoshioka et al. 2010; 2011). Furthermore, the astrocytic reconstitution of the BBB still occurs in the absence of the fibrotic scar (Yoshioka et al. 2010, 2011). Therefore, it is unlikely that the fibrotic scar plays a crucial role in the repair of the damaged CNS tissue.

## Fibrotic scar as an impediment for axonal regeneration

**Fig. 2:**
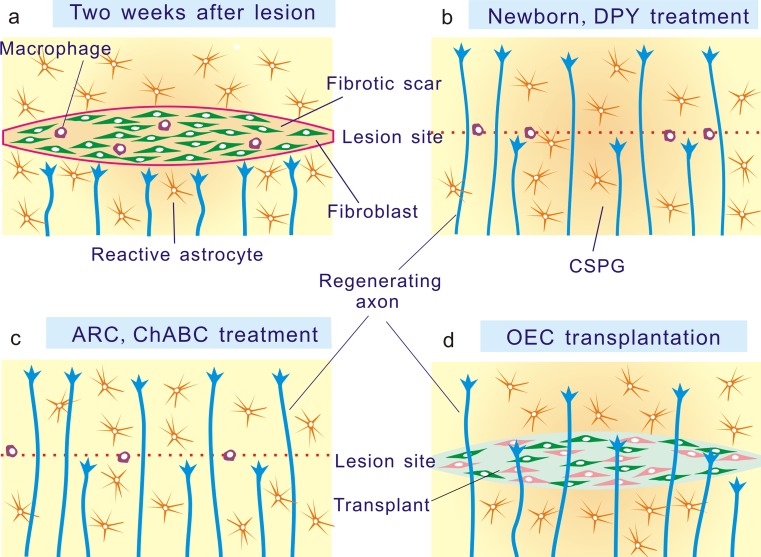
Elimination of the fibrotic scar in the mouse and rat brain has been shown to promote axonal regeneration in a variety of animal models. **a** In injured brain, axons stop at the border of the fibrotic scar and do not regenerate. **b** In neonatal and DPY-treated animals, axons regenerate despite of the presence of glial scar and chondroitin sulfate proteoglycan (*CSPG*) (Stichel et al. 1999a; Kawano et al. 2005). **c** In the hypothalamic arcuate nucleus (*ARC*) and by chondroitinase ABC (*ChABC*) treatment, upregulation of chondroitin sulfate is prevented and axons regenerate (Homma et al. 2006; Li et al. 2007). **d** In olfactory ensheathing cell (*OEC*)-transplanted rats, fibrotic scar is not formed and axons regenerate (Teng et al. 2008)

There are reports that transected axons stop at the border of the fibrotic scar (Fig. 2a; Camand et al. 2004; Stichel and Müller 1994) and fibroblasts have been shown to express various axonal growth-inhibitory molecules including NG2 proteoglycan (Tang et al. 2003), phosphacan (Tang et al. 2003), tenascin-C (Tang et al. 2003), semaphorin 3A (Pasterkamp et al. 1999) and EphB2 (Bundesen et al. 2003). However, since the CNS tissue is rapidly walled off by astrocytes, the ability of the fibrotic scar to present either a physical or molecular obstacle to the regeneration of severed axons depends upon the extent to which the axons come into contact with it.

### DPY treatment (Fig. 2b)

Elimination of the fibrotic scar has been shown to allow axonal regeneration in a variety of animal models (reviewed in Kawano et al. 2007; Klapka and Müller 2006; Fig. 2). The idea of inhibiting the formation of the fibrotic scar was first introduced by the group of Müller (Stichel and Müller 1998; Stichel et al. 1999a, b). Inhibition of Type IV collagen synthesis by administration of DPY into the lesion site prevents the fibrotic scar formation and has been associated with regeneration of postcommissural fornix axons in the injured rat brain (Stichel and Müller 1998; Stichel et al. 1999a, b). This treatment also promoted the regeneration of mouse nigrostriatal dopaminergic axons (Kawano et al. 2005). These results suggest that Type IV collagen is required for the fibrotic response to adult brain injury. Local injection of antibodies against Type IV collagen also suppressed the fibrotic scar formation after the transection of the postcommissural fornix in adult rats (Stichel et al. 1999a). DPY treatment was also applied to the spinal cord injury but was unable to suppress the larger amounts of Type IV collagen that were deposited in this site (Hermanns et al. 2001). Thereafter, treatment with both DPY and cyclic AMP, which inhibits fibroblast proliferation, transiently suppressed the fibrotic scar formation in the injured spinal cord and has been reported to promote regeneration of corticospinal tract axons and recovery of motor function (Klapka et al. 2005).

### Neonatal animals (Fig. 2b)

At earlier stages of development, there are a number of situations in which axons have been reported to regenerate successfully, with recovery of function after injury (for review, see Xu and Martin 1991; Nicholls and Saunders 1996). When nigrostriatal dopaminergic axons are unilaterally transected in mice aged postnatal day 7, dopamine axons regenerate across the lesion site, while they stop and do not extend across the lesion site in mice transected at postnatal day 14 or older (Kawano et al. 2005). Reactive astrocytes bearing CSPGs were increased around the lesion in mice transected at all ages. However, a fibrotic scar containing Type IV collagen deposits was not formed in mice lesioned at postnatal day 7. The fibrotic response is operational in adults but does not occur in wounds of the rat cerebral cortex before 8–10 days after birth (Berry et al. 1983; Maxwell et al. 1990b). Thus, the period of failure of axonal regeneration is correlated with the postnatal development of the Type IV collagen deposition in the lesion site, suggesting that the formation of the fibrotic scar could be a contributory cause of the age-related failure of axonal regeneration in the ascending dopaminergic system.

### ChABC treatment (Fig. 2c)

After injury to the adult CNS, the increase in CSPG around the lesion site is generally believed to constitute a major impediment for axonal regeneration (for review, see Carulli et al. 2005; Höke and Silver 1996; Morgenstern et al*.* 2002; Silver and Miller 2004). It has been reported that degradation of CSPG by injection of ChABC into the lesion site enhances regeneration of nigrostriatal ascending (Moon et al. 2001) and spinal descending (Bradbury et al. 2002) systems. Subsequently, this treatment was shown to suppress the fibrotic scar formation and promote axonal regeneration of the ascending dopaminergic pathway (Li et al. 2007).

### NPY neurons in the hypothalamic arcuate nucleus (Fig. 2c)

Some neurons in the adult mammalian CNS seem to have relatively high capacity to regenerate after transection. An example is the system of neuropeptide Y (NPY) containing neurons in the hypothalamic arcuate nucleus. Since arcuate NPY neurons exert a potent orexigenic function, many experiments have been performed to examine the effect of their destruction by electrolytic or chemical lesions and surgical deafferentation of the projection. Alonso and Privat (1993a) surgically cut NPY axons from the arcuate nucleus and reported axonal regeneration beyond the lesion site. They further found that the astrocytic response in this region differs from other brain regions and suggested that axonal regeneration of arcuate NPY neurons is attributed to the particular organization of the glial scar in this region (Alonso and Privat 1993b).

Administration of gold thioglucose, a neurotoxic glucose analog, to mice increased their body weight and produced a hypothalamic lesion that extended from the ventromedial part of the hypothalamus (Marshall et al. 1955). This treatment transected axons from arcuate NPY neurons but 2 weeks later, they regenerated and extended across the lesion site (Homma et al. 2006). The lesion site was identified by accumulation of reactive astrocytes but a fibrotic scar was not formed, suggesting that the absence of a fibrotic scar may be a permissive factor in the regeneration of the axons of arcuate NPY neurons.

### Transplantation of olfactory ensheathing cells (OECs) (Fig. 2d)

Over the past 10 years, transplantation of OECs into the lesion site of the spinal cord has been shown to promote axonal regeneration and functional recovery (Franklin et al. 1996; Li et al. 1997). In the olfactory bulb, OECs have been described as opening a pathway through the astrocytic covering of the CNS (Raisman 1985). The pathway hypothesis of axon regeneration proposes that nerve fibers will regenerate if they are able to access an aligned pathway of glial cell surfaces (Raisman and Li 2007). OECs transplanted into the lesion site are postulated to provide a pathway for the regeneration of transected axons by opening up the arrangement of the astrocytic processes at the scar interfaces. Possible beneficial effects of OEC transplantation may also include protection of neuronal degeneration, secretion of growth factors, tissue sparing, angiogenesis and remyelination (reviewed in Barnett and Riddell 2004; Radtke et al. 2009). OECs transplanted into injured rat spinal cord were reported to reduce the formation of glial scar (Garcia-Alias et al. 2004; Ramer et al. 2004), while others observed strong astrocytic reaction around the transplanted tissue (Ramón-Cueto et al. 2000; Teng et al. 2008). Although CSPG expression around the lesion was also reported to be reduced by OEC transplantation, neurocan immunoreactivity was unchanged (Garcia-Alias et al. 2004). In other studies, high levels of CSPG expression after lesion were not affected by cell transplantation (Jones et al. 2003; Ramer et al. 2004; Teng et al. 2008).

In rats transected with a nigrostriatal dopaminergic pathway, few dopaminergic axons extended across the lesion at 2 weeks after the transection. When OECs were transplanted into the lesion site, many dopaminergic axons regenerate to extend over the lesion. In these animals, the deposition of Type IV collagen and the fibrotic scar formation were not detectable in the lesion site (Teng et al. 2008).

## Mechanism of fibrotic scar formation

Meningeal cells make an important contribution to the formation of the fibrotic scar (reviewed by Shearer and Fawcett 2001), although a recent study has reported that a specific subtype of vascular pericytes also gives rise to scar-forming stromal cells (Göritz et al. 2011). In rat spinal cord lesions, where the dura mater was left intact, fibroblastic infiltration to the lesion site was much reduced (Fernandez and Pallini 1985) and axons regenerated beyond the lesion (Seitz et al. 2002). Duraplasty with cadaveric rat dura mater allograft over the injury site of the rat spinal cord reduced fibrotic scarring at the lesion site (Iannotti et al. 2006).

In the intact brain, meningeal fibroblasts are stable or quiescent, i.e. they express low levels of ECMs and do not actively proliferate. When the brain is damaged, meningeal cells are stimulated; they gain mobility to migrate to the lesion site where they actively proliferate and produce the ECMs. Although the exact mechanism underlying the stimulation of meningeal fibroblasts after traumatic injury is not yet known, it is highly likely that the bleeding in the CNS is involved in the activation of meningeal fibroblasts. When the transection of spinal cord was performed by a sharp knife so that bleeding was minimized, a fibrotic scar was not formed (Iseda et al. 2003).

After breakdown of the BBB, infiltrating leukocytes and CNS-resident microglia secrete various cytokines and growth factors that are involved in the inflammatory response (reviewed in Donnelly and Popovich 2008; Lenzlinger et al. 2001; Merrill and Benveniste 1996) and they are increasingly expressed with characteristic spatiotemporal patterns. Expression of pro-inflammatory cytokines including interleukin 1α, 1β and 6, tumor necrosis factor α and leukemia inhibitory factor are acute and only transient after CNS injury, steeply increase by 6 h, reach a peak at 12 h and decline by 24 h after injury (Bartholdi and Schwab 1997; Nakamura et al. 2003; Streit et al. 1998). In contrast, expression of TGF-β1, an anti-inflammatory cytokine, is delayed and continuous after CNS injury. TGF-β1 expression increases around the lesion site during the period of fibrotic scar formation, increasing from 2 days, reaching a peak at 4 days and declining, but with still enhanced levels, at 2 weeks after CNS injury (Lagord et al. 2002; Nakamura et al. 2003; Semple-Rowland et al. 1995; Streit et al. 1998). Considering that TGF-β1 is a potent fibrogenic factor that enhances its proliferation and ECM production of fibroblasts (Ignotz and Massague 1986; Moses et al. 1987), it seems apparent that TGF-β1 is involved in the activation of meningeal fibroblasts after CNS injury.

### Localization of TGF-β receptors in injured CNS

The biological action of TGF-β1 is mediated through binding to both type I and type II TGF-β receptors (TRI and TRII). TRII binds to its specific ligand but TRI requires the presence of bound TRII to interact with TGF-βs (Wrana et al. 1992). As a result, TRI and TRII are co-localized in many cases on same cells. In adult normal mouse brains, the expression of TRI and TRII is at a very low level, while it is upregulated after traumatic CNS injury (Fee et al. 2004; McTigue et al. 2000) and in multiple sclerosis lesion (de Groot et al. 1999). After CNS lesioning, TRI and TRII are expressed on neurons and astrocytes (de Groot et al. 1999), endothelial cells of the blood vessels (de Groot et al. 1999; Fee et al. 2004), macrophages (de Groot et al. 1999; McTigue et al. 2000) and fibroblasts (Komuta et al. 2010).

Fibroblasts bearing receptor mRNAs are first detected in the meninges and around blood vessels at a 1 day after injury. Three days after the injury onward, fibroblasts with receptor messages increase in the lesion site and the majority of fibroblasts in the fibrotic scar express receptor mRNAs. Furthermore, TRI and TRII are also expressed in fibroblasts along the migratory pathway from meninges to the lesion site (Komuta et al. 2010), indicating that the meningeal fibroblasts that form the fibrotic scar are a major target of TGF-β1 upregulated after CNS injury.

### The role of TGF-β on the scar formation

The manipulation of TGF-β signaling in the injured CNS modulates formation of the fibrotic scar in the lesion site. The administration of TGF-β1 to injured CNS increases the deposition of ECMs in the lesion site (Hamada et al. 1996; Logan et al. 1994), while antibodies to TGF-β1 and TGF-β2 and the endogenous TGF-β inhibitor decorin, a small leucine-rich CSPG, conversely reduce the size of fibrotic scar (Logan et al. 1994, 1999a, b), which suggests the involvement of TGF-βs in the formation of fibrotic scar.

Receptor activation by TGF-βs leads to phospholylation of Smad2 and Smad3 by the TRI (reviewed in Derynck and Zhang 2003; Heldin et al. 1997). Phospholylated Smads interact with a diverse array of transcription factors to bring about TGF-β-regulated transcription (Feng and Derynck 2005; ten Dijke and Hill 2004). When LY-364947, a small molecule inhibitor of TRI, is continuously infused in the lesion site of mouse brain, the fibrotic scar formation is completely suppressed (Yoshioka et al. 2011). In Smad3 null mice, expression of fibronectin and laminin was also reduced (Wang et al. 2007). These results indicate that inhibition of TGF-β signaling is likely to suppress the formation of the fibrotic scar.

**Fig. 3:**
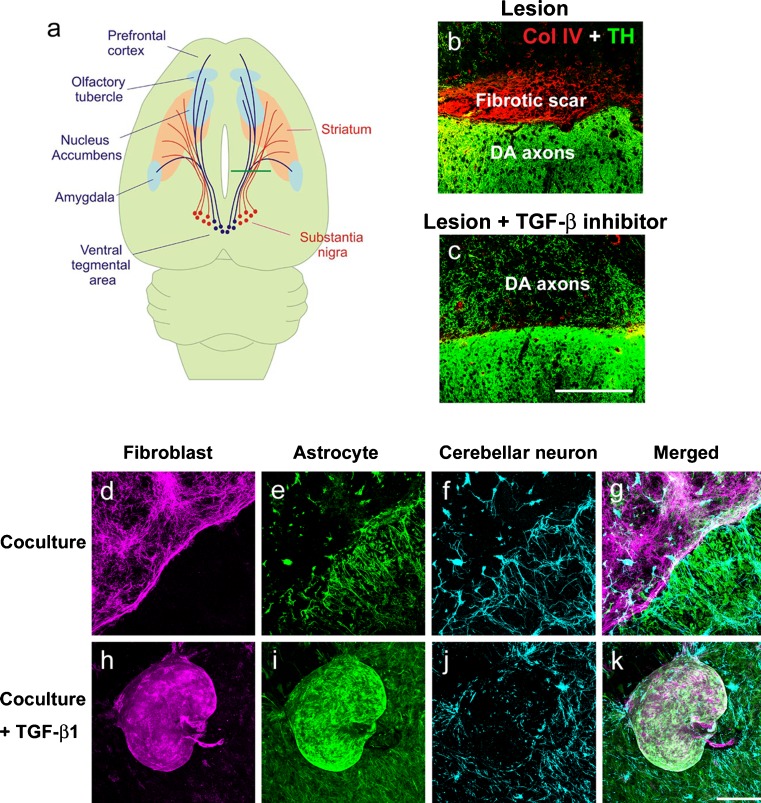
Role of TGF-β on the formation of fibrotic scar which inhibits axonal regeneration. **a** Schematic drawing of the transection of mouse brain (Kawano et al. 2005). Ascending dopaminergic axons which arise from the substantia nigra and ventral tegmental area project to the telencephalic structures are cut at the proximal part of the striatum (*green line*) with a knife of 2 mm width. **b** The fibrotic scar containing dense Type IV collagen (*Col IV*) deposits (*red*) is formed in the lesion site 2 weeks after injury and transected tyrosine hydroxylase (*TH*)-immunoreactive dopamine (*DA*) axons (*green*) stop at the fibrotic scar. **c** Continuous injection of the inhibitor of TGF-β, LY-364947 into the lesion site completely suppresses the fibrotic scar formation and promotes axonal regeneration (Yoshioka et al. 2011). **d–k** In vitro model of the lesion scar (Kimura-Kuroda et al. 2010). **d–g** Meningeal fibroblasts (magenta) and cerebral astrocytes (*green*) form separate colonies in coculture. Cerebellar neurons grow better on astoricytes than on fibroblasts. **h–k** When TGF-β1 is added to the coculture, cells aggregate to form a fibrotic scar-like cluster which repels neurites of cerebellar neurons (*blue*). *Scale bars* (**b**, **c**) 200 μm, (**d**–**k**) 100 μm

The effect of the inhibition of TGF-β signaling on axonal regeneration is controversial. The majority of authors did not find the regeneration of transected axons by the inhibition of TGF-β signaling despite the reduction of the scar tissue (Logan et al. 1994, 1999a, b; Moon and Fawcett 2001; King et al. 2004). In contrast, Davies et al. (2004) demonstrated that decorin suppressed the deposition of CSPGs in the lesion site and promoted axon growth from transplanted sensory neurons, although axonal regeneration of intrinsic neurons was not described. In mice with unilateral transection of the nigrostriatal dopaminergic pathway, dopaminergic axons scarcely extended beyond the fibrotic scar, while they regenerated over the fibrotic scar-free lesion site in mice treated with the inhibitor of TRI, LY-364947 (Fig. 3a–c) (Yoshioka et al. 2011).

### Microtubule stabilization

Microtubule dynamics regulate key processes during scarring, including cell proliferation, migration and secretion of ECMs (Liu et al. 2005; Westermann and Weber 2003). Moderate microtubule stabilization with Taxol reduces the formation of fibrotic scar and allows axonal regeneration and functional recovery after spinal cord injury (Hellal et al. 2011). Taxol treatment hinders Smad2 trafficking in TGF-β signaling, reduces the TGF-β1-stimulated production of fibronectin in cultured meningeal cells and impairs TGF-β1-stimulated migration, thus reducing fibrotic scarring after spinal cord injury.

### In vitro model of the CNS lesion site

Attempts to reproduce the regeneration-inhibitory property of the CNS lesion site in vitro have been repeatedly performed using coculture of cerebral astrocytes and meningeal fibroblasts (Abnet et al. 1991; Hirsch and Bahr 1999; Struckhoff 1995). When cocultured, the two kinds of cells separately form flat colonies and rarely overlap each other (Fig. 3d–g). Such an interface may be considered as providing an in vitro model of the glia limitans (Abnet et al. 1991), which is the lining of astrocytic processes surrounding the fibrotic scar in the CNS lesion site (Berry et al. 1983). In a similar co-culture system, meningeal fibroblasts express moderate amounts of the axonal growth-inhibitory molecules NG2 proteoglycan, versican and class 3 semaphorins, while astrocytes express the axonal growth-promoting molecules N-cadherin and laminin (Shearer et al. 2003). More recently, Wanner et al. (2008) demonstrated that an addition of meningeal fibroblasts to cultured astrocytes enhanced expression of GFAP, the CSPGs phosphacan and neurocan and tenascin-C in astrocytes. Modeling traumatic injury by mechanically stretching the co-culture did not further activate astrocytes. In these co-cultures, major characteristics of the fibrotic scar, i.e., proliferation of fibroblasts, dense accumulation of ECMs and high expression of axonal growth-inhibitory molecules are not observed (Shearer and Fawcett 2001; Shearer et al. 2003; Wanner et al. 2008).

Addition of TGF-β1 to the coculture of cerebral astrocytes and meningeal fibroblasts resulted in enhanced proliferation of fibroblasts and the formation of cell clusters, which consisted in fibroblasts inside and surrounded by astrocytes (Kimura-Kuroda et al. 2010). The cell cluster in culture densely accumulated the ECMs and axonal growth-inhibitory molecules similar to the fibrotic scar. The expression of Type IV collagen, NG2, CS, phosphacan, semaphorin 3A, EphB2 and tenascin-C in fibroblasts and neurocan, phosphacan, ephrin-B2 and tenascin-C in astrocytes, was greatly enhanced in the cluster induced by TGF-β1. In this coculture, the neurite outgrowth of cerebellar neurons was promoted on astrocytes, inhibited on fibroblasts and remarkably suppressed on the cluster (Fig. 3h–k). In this aspect, this culture system mimics a CNS lesion site and may provide a model to analyze the inhibitory property in the lesion site of CNS (Kimura-Kuroda et al. 2010).

In peripheral tissues, TGF-β1 is known to affect various kinds of mesodermal cells to induce physiological and pathological fibrosis (for review, see Cutroneo 2007; Wynn 2008; Kisseleva and Brenner 2008). A variety of cultured mesenchymal cells from kidney, heart, lung, liver, spleen and skin form aggregates and actively produce ECMs when stimulated by TGF-β1 (Xu et al. 2007).

## Concluding remarks

Virtually all lesions of the CNS open the BBB, whether from outside, through the pia, or from blood vessels within the CNS tissue. The rapid post-injury response by the astrocytes serves to seal this breach in the BBB. Subsequently, there is a fibroblastic reaction around the astrocytes. Astrocytes and fibroblasts interact to form an organized tissue (the ‘scar’). By sealing off the damage and restoring the BBB, the astrocytic reaction is protective. Both astrocytes and fibroblasts express abundant axon-repelling molecules. Suppression of TGF-β signaling has been shown to be an effective tool for preventing formation of the fibrotic scar and has been reported to promote axonal regeneration without detrimental effects on the sealing process of damaged CNS.
